# Positive ADHD Scores are Associated With Higher Screen Time and Anxiety Symptoms in Medical Students: Cross-sectional Study

**DOI:** 10.62641/aep.v53i3.1892

**Published:** 2025-05-05

**Authors:** Julia Sader Neves Ferreira, Roberta Molaz da Silva, Carolina Fauzi Hamuche, Rafael Bonfim do Nascimento, Ana Paula Ribeiro, Saulo Gil, Lucas Melo Neves

**Affiliations:** ^1^Medicine Graduation Department, Santo Amaro University, 04743-030 São Paulo, Brazil; ^2^Physical Activity, Sport and Mental Health Laboratory (LAFESAM), Department of Physical Education, São Paulo State University (UNESP), 13506-900 Rio Claro, Brazil; ^3^Faculty of Medical Sciences of Santa Casa de São Paulo, 01224-001 São Paulo, Brazil; ^4^Post-Graduate Program in Health Sciences, Santo Amaro University, 04743-030 São Paulo, Brazil; ^5^Bipolar Disorder Program (PROMAN), Department of Psychiatry, University of São Paulo Medical School, 05508-220 São Paulo, Brazil

**Keywords:** student health, cognition, attention, screen time, ADHD, anxiety

## Abstract

**Background:**

Attention deficit hyperactivity disorder (ADHD) refers to a set of symptoms, such as an inability to sustain attention, hyperactivity, and impulsivity, with a prevalence of 2.0% for the general population. Approximately 2.7% of American medical students report having some form of disability, with ADHD emerging as the most frequently self-disclosed condition. Medical students with a positive ADHD Self-Report Scale (ASRS) score present more depression symptoms in comparison with those with a negative ASRS score. Previous studies suggest that a low amount of time spent in physical activity and a high amount of time spent in sedentary behavior were associated with mental disorders (e.g., anxiety and depression). However, information in the literature on this association with symptoms of ADHD is limited, particularly in medical students.

**Methods:**

In this cross-sectional study, we investigated a sample of medical students aged 18 years or older. Individuals diagnosed with ADHD were excluded. Participants completed an online survey, which included questions about demographic and academic experiences, the ADHD Self-Report Scale, the International Physical Activity Questionnaire (IPAQ), and the Beck Anxiety Inventory (BAI). Statistical analysis was conducted using the SPSS 22 program, with a significance threshold of *p* = 0.05.

**Results:**

Out of ninety-nine medical students included, forty individuals (40.4%), demonstrated positive ASRS scores, suggesting a risk for ADHD. After dividing the participants into groups according to their ASRS scores (negative or positive ASRS), the Mann-Whitney comparison revealed that the negative ASRS group exhibited lower daily screen time (9.0 vs 12.0 hours per day; *p* < 0.01) and reduced anxiety symptoms (8.0 vs 16.0 points; *p* < 0.01) compared to the positive ASRS group. Furthermore, the linear multiple regression analysis indicated that screen time was a predictor of the ASRS score.

**Conclusion:**

In a sample of medical students, the current study showed a prevalence of 40.4% of positive ASRS. The results suggest that medical students with a positive ASRS score have higher screen time, as well as more symptoms of anxiety. In addition, we found that screen time was a significant predictor of scores in the ASRS.

## Introduction 

Attention deficit hyperactivity disorder (ADHD) encompasses a range of symptoms, including difficulties in maintaining attention, hyperactivity, and impulsivity [1]. This medical condition has a prevalence of 2.0% for the general population [2]. In the context of medical students, it is noteworthy that 2.7% of American medical students report having some form of disability, with ADHD emerging as the most frequently self-disclosed condition, affecting 30.0% of students with a self-disclosed disability [3].

The diagnosis of ADHD requires a thorough evaluation of current and historical symptoms, functional impairment, and a complete family, gestational, and developmental history [4]. According to the eleventh edition of the International Classification of Diseases (ICD-11) [5], the criteria for ADHD include persistent inattention, hyperactivity-impulsivity, or both. The onset typically occurs in early to mid-childhood, with symptoms impacting academic, occupational, and social functioning. Despite established criteria, ADHD is frequently underdiagnosed and undertreated in clinical practice, potentially due to the intricate evaluation processes [2, 6]. In this scenario, alternative strategies are needed to identify symptoms associated with ADHD, particularly in a non-clinical setting.

The Adult ADHD Self-Report Scale (ASRS) is the original version of the ASRS tool used to assess symptoms of ADHD [7]. The six-question ASRS Screener or ASRS version 1.1, is a subset of the items from the ASRS. This version was shown to be a reliable and valid scale for evaluating symptoms of ADHD in adults, and presents high internal consistency and high concurrent validity with the full version of ASRS [8]. While examining ADHD symptoms across diverse populations is crucial, in the current study we underscore its importance for medical students.

The university phase aligns with the developmental stage of emerging adulthood (ages 18–25), characterized by heightened independence relative to adolescence, while accompanied by incomplete cognitive maturation [9]. This period is marked by a notable reduction in parental support, an increase in temptations and distractions for students [10], and a significant demand for learning, typical of medical courses.

In this sense, Galván-Molina and collaborators [11] identified a significant percentage (28.0%) of medical students with possible ADHD considering a positive ASRS 1.1 score (if the score ≥4, the screening is positive). Interestingly, medical students with a positive ASRS 1.1 score also had a higher proportion of depression in comparison with those with a negative score on the six-question ASRS [11]. These findings suggest that the ASRS 1.1 may serve as an effective screening tool to detect ADHD symptoms (not for establishing diagnoses) in medical students.

Significantly, an additional inquiry pertinent to mental health in medical students pertains to their lifestyle choices. The various routines and activities undertaken by medical students are well-documented in their potential to influence both physical and mental health. Notably, excessive screen time and prolonged periods of sedentary behavior are both acknowledged for their detrimental effects on a range of health outcomes [12, 13]. In accordance with a study conducted by Liebig *et al*. [14], medical students allocate approximately seven hours per day to screen-based activities, potentially influencing their mental health. Previous studies have indicated that reduced physical activity and increased sedentary behavior are linked to adverse mental health outcomes, such as anxiety and depression symptoms [15, 16, 17]. However, the existing literature offers limited insights into this association concerning ADHD symptoms, particularly among medical students. In this context, it is reasonable to assume that medical students with higher symptoms of ADHD tend to engage in less favorable lifestyle choices, characterized by reduced physical activity and increased screen time, than their counterparts with fewer symptoms of ADHD.

Therefore, the objective of the current study is as follows: (1) to investigate the prevalence of medical students exhibiting positive ASRS 1.1 scores; (2) to compare those with positive ASRS 1.1 scores with their counterparts with negative ASRS 1.1 scores regarding screen time, sedentary behavior and physical activity, and anxiety symptoms; (3) to verify if screen time, time of physical activity, and anxiety symptoms may be related to ASRS scores. We hypothesized that a significant number of medical students would exhibit positive ASRS scores, elevated screen time, sedentary behavior, and anxiety symptoms, and reduced physical activity.

## Methods

### Study Design and Participants

The present cross-sectional study received approval from the Santo Amaro University Ethics and Research Committee (approval number: 5.496.734). It was conducted at the Santo Amaro University, located in São Paulo, Brazil. The data were collected in April 2022. Participants were recruited by means of messaging applications and social media platforms, followed by an online survey using Google forms® (Google LLC, Mountain View, CA, USA)—presented in **Supplementary File**, which included a consent form, a questionnaire to gather information on demographic and academic characteristics, and self-reported questionnaires on ADHD symptoms (ASRS 1.1) [7, 8], sedentary behavior and physical activity time (International Physical Activity Questionnaire, IPAQ) [18], and anxiety symptoms (Beck Anxiety Inventory, BAI) [19].

#### Criteria Inclusion and Exclusion

The study included medical students: (a) enrolled in any of the 1st to 12th semesters of Brazilian medical school programs (Basic cycle—1st to 4th semester; Clinic cycle—5th to 8th semester; Internship—9th to 12th semester); (b) 18 or more years of age. Participants with a prior ADHD diagnosis were excluded from the research.

#### Sample Size Calculation

The sample size was determined using G-Power software (version 3.1.2, Universitat Kiel, Kiel, SH, Germany), considering a total sample size of 900 students, and Exact - Proportions: Inequality, with two dependent groups (Odds ratio = 1.28, β/α ratio = 0.95). The sample size calculations indicated a minimum of 96 students (α err prob = 0.21; β err prob = 0.20; Power (1-β err prob) = 0.80).

### Data Sources/Measurement

#### Attention Deficit and Hyperactivity 

The instrument utilized to assess symptoms of ADHD was the Adult ASRS - Version 1.1 (ASRS 1.1) [7], designed as a self-report scale for the purpose of screening ADHD symptoms in World Mental Health surveys conducted by the World Health Organization (WHO) [7]. The ASRS 1.1 presents satisfactory internal consistency (Cronbach’s alpha 0.88) and intraclass correlation coefficient (ICC) (0.84) compared to the original ASRS [8]. Furthermore, the concise nature and ability to differentiate between Diagnostic and Statistical Manual-IV (DSM-IV) cases and non-cases make the ASRS Screener an attractive tool for community-based epidemiological studies, as well as clinical outreach and case identification efforts [7].

The ASRS consists of six items that capture comprehensive data regarding attention difficulties and hyperactivity levels experienced during the preceding six-month period. For the final interpretation, we considered the proposal from Kessler and collaborators. Each question in the study presents the participant with five alternatives. The options include: “never”, “rarely”, “sometimes”, “often”, and “very often”. To establish a potential diagnosis of ADHD, it was necessary for the participant to have four or more answers of “sometimes”, “often”, or “very often” for the third question and choose “often” or “very often” for the fourth through sixth questions [7].

#### International Physical Activity Questionnaire

The International Physical Activity Questionnaire (IPAQ) is a widely used tool for assessing physical activity levels in individuals. This questionnaire was specifically designed as a tool for internationally monitoring physical activity and sedentary behavior. The instrument presents strong reliability, as indicated by the Spearman correlation coefficient of 0.80, both in terms of its internal consistency and test-retest reliability [18]. Noticeably, the usage and validation of the IPAQ have been conducted in the Brazilian population [20].

In brief, the IPAQ comprises a set of eight inquiries pertaining to physical activity and sedentary behavior. The following inquiries evaluate the subject’s weekly regimen, with specific emphasis on the frequency and length of walking, as well as moderate or vigorous physical activity. The time spent engaging in walking and Moderate Vigorous Physical Activity (MVPA) is quantified in terms of minutes per week. Sedentary behavior data are presented as hours per day.

#### Screen Time Assessment

The evaluation of screen time was estimated considering: (i) television consumption, which includes digital versatile discs (DVDs), television shows, series, and so on; (ii) computer or tablet usage; and (iii) video game play. In addition, the amount of time individuals spent using their smartphones was used as an indicator of smartphone time use. To access these data, the user is required to browse to the configuration settings of their smartphone. This may be accomplished by choosing either the “Settings” option, followed by “Digital Wellbeing and Parental Controls”, or alternately, by selecting “Settings” and then “Usage time”.

#### Anxiety Symptoms Assessment

The assessment of anxiety symptoms was conducted utilizing the Beck Anxiety Inventory (BAI) [19]. This scale presents high reliability, as seen by the Cronbach’s alpha coefficient of 0.95 and the test-retest reliability of Pearson’s r ranging from 0.73 to 0.96, including a validated adaptation to the Brazilian population, where there is a significant prevalence of anxiety among the general population [21].

The BAI is composed of 21 multiple-choice questions, each of which offers four possible answers (ranging from 0–3), which can lead to final scores of 0 to 63 points. Scores of 0 to 21 indicate the absence of or low anxiety, 22 to 35 indicate moderate anxiety, and scores of 36 and above indicate potentially concerning levels of anxiety [19].

### Statistical Analysis

The participants in the study were separated into two groups based on their scores in the ASRS. The first group, referred to as the negative ASRS group, consisted of individuals who scored below 4 points on the scale, indicating no diagnostic probability of ADHD. The second group, referred to as the positive ASRS group, consisted of individuals who scored 4 points or above on the scale, indicating a potential diagnosis of ADHD.

The normality and equality of variance of the data were assessed using the Shapiro-Wilk and Levene’s tests, respectively. A chi-square test was used to compare the groups (positive ASRS vs. negative ASRS) for each categorical variable. For the continuous variables such as age, weight, and height, the *t*-test was used for between-group comparisons (means ± standard deviation (SD)). For others continuous variables including screen time, anxiety symptoms, walking time, moderate to vigorous physical activity time, and sedentary behavior time, a Mann-Whitney test was used for between-group comparisons (median and Q1–Q3).

Crude and adjusted (by age [as continuous variable], body weight [as continuous variable], ethnicity [white, black, and pardo], and sex [male or female]) linear regression models were utilized to verify possible associations between screen time and anxiety symptoms with ASRS score. Beta coefficients were calculated along their corresponding 95% confidence interval (CI) (95% CI). The level of significance was established at a threshold of *p* < 0.05. The statistical analysis was conducted using SPSS 22 software (IBM SPSS Statistics for Windows, Version 22.0, Chicago, IL, USA).

## Results 

A total of ninety-nine medical students participated in the study. Among the medical students included, a notable proportion, 40.4% (n = 40), exhibited positive ASRS scores (scoring ≥4). Participants were divided into two groups based on their negative or positive ASRS scores. There were no significant variable differences between the groups, as shown in Table 1.

**Table 1.** **Characteristics of the sample**.

Variable	All	Negative ASRS	Positive ASRS	*p*-value	*t*-value	Chi-square
	(n = 99)	(n = 59)	(n = 40)			
General information						
Age (years) &	24 ± 5	24 ± 5	24 ± 4	0.97	0.03	–
Weight (kg) &	66.7 ± 12.1	65.0 ± 12.3	68.2 ± 12.3	0.26	–1.34	–
Height (cm) &	166 ± 9	165 ± 9	167 ± 8	0.19	–1.07	–
Woman (n - %) #	78 - 79	47 - 80	31 - 78	0.80	–	0.067
Period of course #						
Basic cycle (n - %)	18 - 18	9 - 15	9 - 22	0.55	–	1.18
Clinic cycle (n - %)	49 - 50	29 - 49	20 - 50			
Internship (n - %)	32 - 32	21 - 36	11 - 28			
Ethnicity #						
White (n - %)	87 - 88	51 - 88	35 - 86	0.92	–	0.15
Pardo^1^ (n - %)	11 - 11	7 - 11	4 - 10			
Yellow (n - %)	1 - 1	1 - 2	1 - 2			
Consumption of legal or illegal drugs #						
Cigarettes use (n - %)	13 - 13	8 - 14	5 - 13	0.878	–	0.023
Alcohol use (n - %)	70 - 71	38 - 64	32 - 80	0.09		2.80
Cannabis use (n - %)	14 - 14	8 - 14	6 - 15	0.84		0.04

Legend: ^1^ = Pardo is the exact term used in Brazilian Portuguese, meaning “mixed ethnicity”, according to the Brazilian Institute of Geography and Statistics. n = number of subjects. Basic cycle = 1st to 4th semester; Clinic cycle = 5th to 8th semester; Internship = 9th to 12th semester. & = *t*-test; # = chi-square values. ASRS, ADHD Self-Report Scale.

**Fig. 1.**
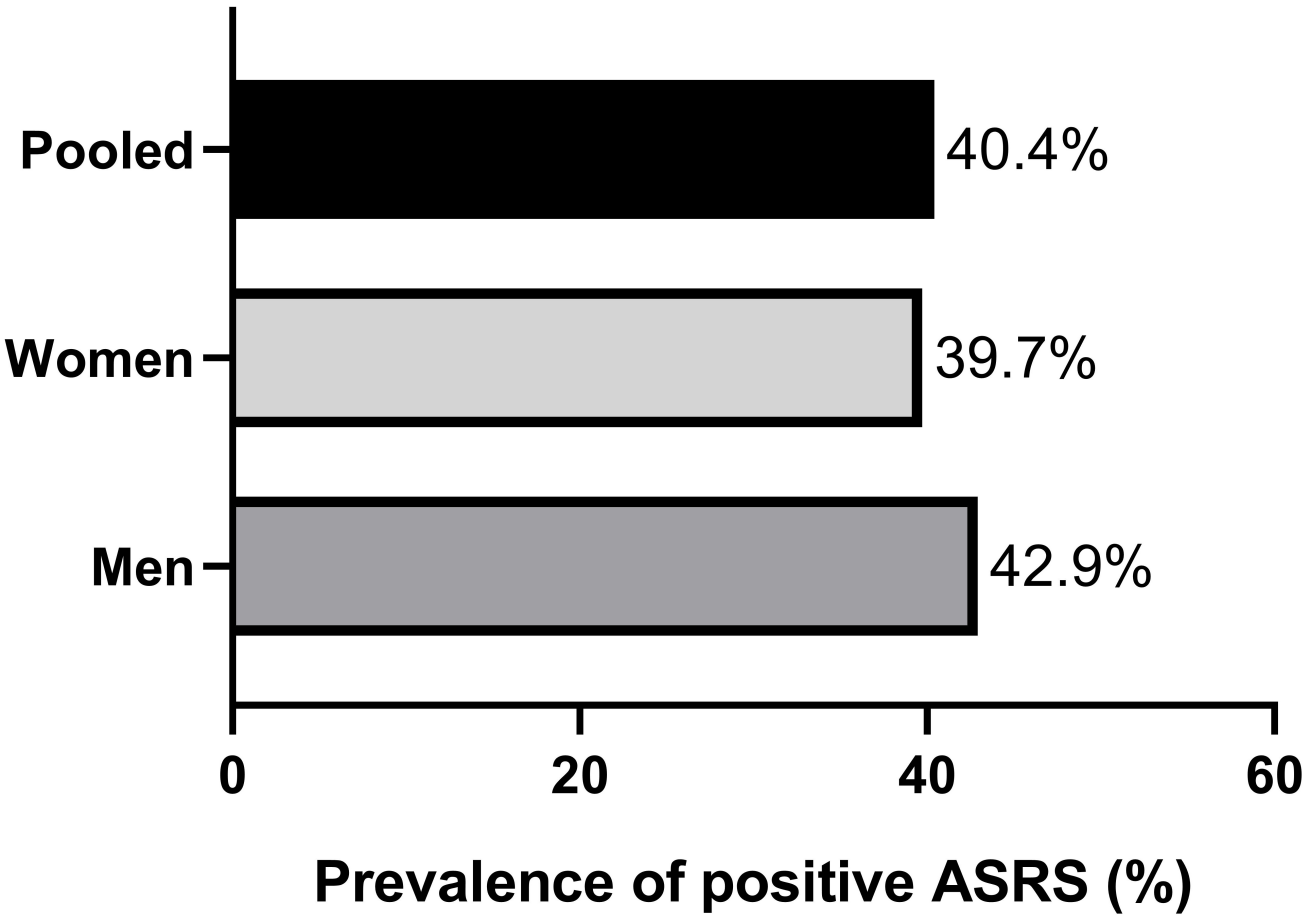
**Prevalence of positive attention deficit and hyperactivity disorder self-report scale by sex**. Legend: ASRS, ADHD Self-Report Scale.

The prevalence of positive ASRS scores was similar between sexes (39.7% and 42.9% for women and men, respectively, *p* = 0.66). Fig. 1 details the prevalence of positive ASRS scores by sex.

**Fig. 2.**
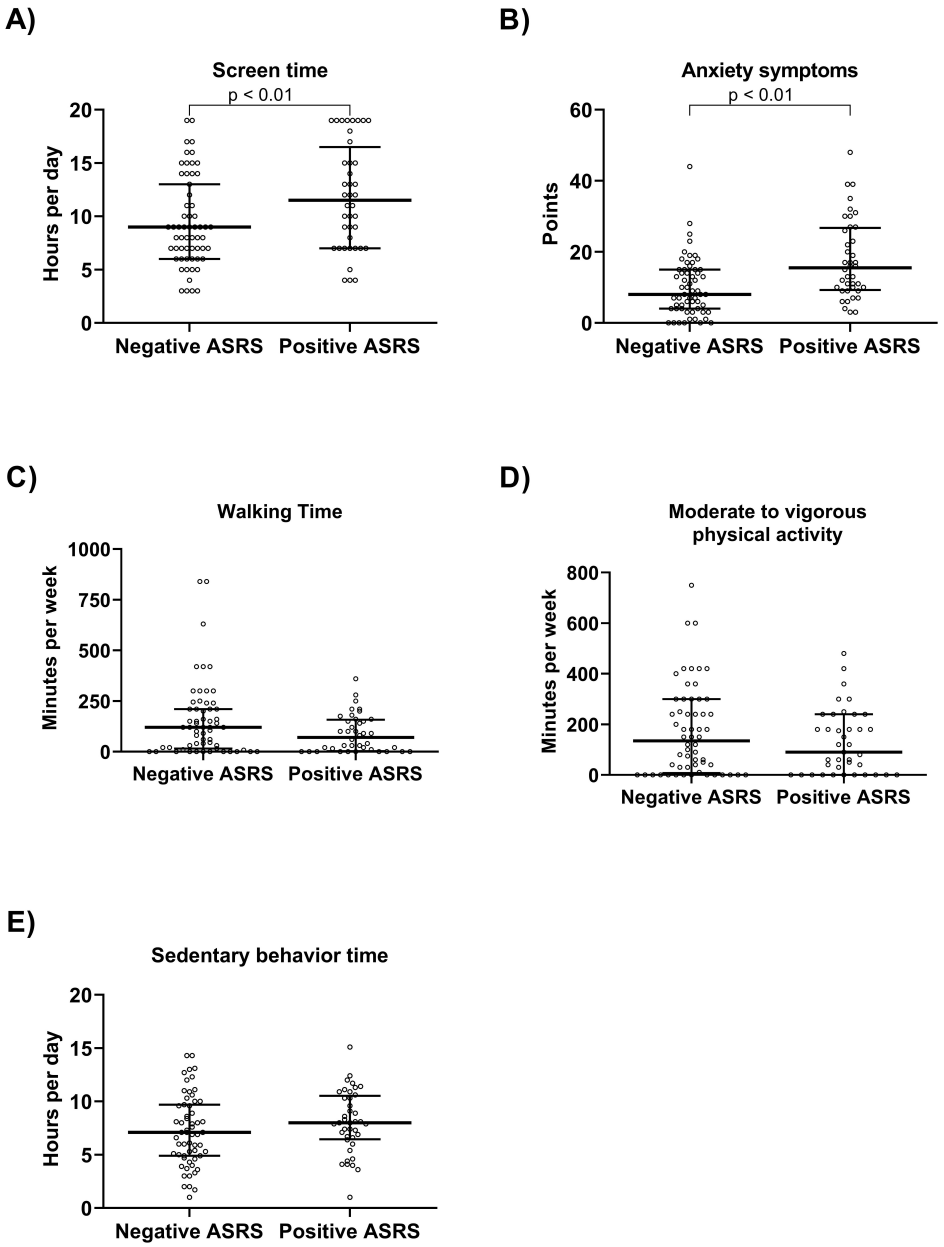
**Comparison between groups Negative and Positive ADHD Self-Report Scale (ASRS)**. Daily screen time (A), Anxiety symptoms (B), Walking Time (C), Moderate Vigorous Physical Activity time - MVPA Time (D), Sedentary Time (E). Data presented as median and Q1–Q3.

Fig. 2 provides the comparisons between groups. To summarize, the negative ASRS group, compared to the positive ASRS group, exhibited lower daily screen time (Panel A—9.0 vs. 12.0 hours per day; 95% CI = 0.80 to 4.00; *p* < 0.01) and reduced anxiety symptoms (Panel B—8.0 vs. 16.0 points; 95% CI = 3.0 to 10.0; *p* < 0.01).

There were no significant differences observed between the Negative and Positive ASRS groups in terms of the variables Walking Time (Panel C—120 vs. 70 minutes per day; 95% CI = –85.0 to 0.0; *p* = 0.11), MVPA (Moderate Vigorous Physical Activity) Time (Panel D—135 vs. 90 minutes per day; 95% CI = –80.0 to 20.0; *p* = 0.34), and Sedentary Time (Panel E—7.1 vs. 8.0 hours per day; 95% CI = –0.4 to 2.3; *p* = 0.15).

The crude linear regression model (Table 2) showed a positive association between screen time and ASRS score (β = 0.13; 95% CI = 0.05–0.21; *p* < 0.01). These associations remained statistically significant after adjustments for covariates (age, body weight, ethnicity, and sex (β = 0.12; 95% CI = 0.03–0.20; *p* < 0.01)). In contrast, the crude and adjusted linear regression models demonstrated that screen time is not a statistically significant predictor of anxiety symptoms (both *p* > 0.05).

**Table 2.** **Linear regression analyses of the association between screen time and the ADHD Self-Report Scale (ASRS) and Beck Anxiety Inventory (BAI)**.

Outcome	Unadjusted model			Adjusted model ^a^		
	β	95% CI	*p*-value	β	95% CI	*p*-value
ASRS	0.13	0.05–0.21	<0.01	0.12	0.03–0.20	<0.01
BAI	0.10	–0.35–0.54	0.66	0.04	–0.43–0.51	0.86

Adjusted model ^a^ = adjusted by age (as continuous variable), body weight (as continuous variable), ethnicity (white, black, and pardo), and sex (male or female). CI, confidence interval.

## Discussion

The current cross-sectional study aimed to examine the prevalence of positive ADHD Self-Report Scale (ASRS) scores among medical students and to compare positive and negative ASRS groups in terms of screen time, sedentary behavior (SB) time, physical activity (PA) time, and anxiety symptoms, as predictors of ASRS scores. The main results revealed a high prevalence (men: 42.9%; women: 39.7%; both: 40.4%) of medical students exhibiting positive ASRS scores. Additionally, the group with positive ASRS scores presented higher screen time as well as more anxiety symptoms than their counterparts with negative ASRS scores. Furthermore, screen time was an independent and significant predictor of ASRS scores.

The observed prevalence of positive ASRS in the current study (40.4%) surpasses that reported in previous research (28.0%) among medical students [11], as well as the naturally occurring prevalence (6.1%) of an ADHD diagnosis in medical students [22, 23]. Although screening scales present inherent limitations, which could account for the disparity in prevalence rates between screening and diagnostic assessments, research has shown that the transition from high school to university can negatively impact students who were previously high-functioning and not diagnosed with ADHD during their high school years [24]. In basic terms, it can be observed that these individuals exhibit elevated stress levels and experience greater challenges in managing their symptoms, causing heightened impairment as a result of their previously undetected ADHD [24]. This finding explains, at least partially, the notably high prevalence of positive ASRS scores in the present study.

Prior studies demonstrated a correlation between screen time and attention problems, such as the intensification of symptoms associated with attention-deficit [25, 26]. The persistent engagement, as observed in the context of smartphone usage (auditory and tactile stimuli generated by notifications or vibrations), along with the constant stream of updates from multiple sources, may contribute to the excessive utilization of social networks among individuals who are vulnerable to distraction [27, 28]. Furthermore, there has been an increasing incidence of laptop and tablet use, as indispensable equipment in university courses. These devices are frequently employed for a range of academic tasks, such as note-taking, performing research, and engaging in additional intellectual activities [29].

In the positive ASRS group, it was anticipated that there would be more prominent manifestations of anxiety symptoms. Anxiety frequently co-occurs with ADHD [4]. Additionally, de Souza *et al*. [16] and Santana *et al*. [15] found higher levels of anxiety symptoms among medical students when compared to students in other academic areas [16]. A meta-analysis conducted by Pacheco *et al*. [30] revealed a substantial prevalence of anxiety among medical students, with a rate of 32.9%, based on data from 59 studies. Moreover, Twenge *et al*. [31] revealed positive correlations between screen time and mental health problems. The concurrent relationship between ASRS scores and anxiety symptoms, as well as ASRS scores and screen time, highlights a potential link between mental health and screen time among medical students.

Although our aim is not to elucidate the cerebral mechanisms behind ADHD and anxiety symptoms, prior research indicates a correlation between the two, attributed to intricate dopaminergic gating disruptions in the ventral striatum and nucleus accumbens, modulated by the hippocampus and amygdala [32]. Patients with ADHD and anxiety exhibit neurodevelopmental abnormalities in the brain, such as diminished volumes in the supramarginal and pre/postcentral gyri [33], as well as reduced volumes in the basal ganglia and insula [34]. Significant data indicate that excessive screen time, especially during brain development, correlates with alterations in brain size [35]. When considered collectively, future research that investigates the potential mechanisms of screen time as a potential factor that exacerbates anxiety and ADHD symptoms is pertinent.

The strengths of the present study are primarily associated with the characteristics of the sample. In contrast to previous research, which predominantly focused on younger age groups, for example, adolescents [36], the present study comprised university students, specifically those enrolled in medical programs. This group is of significant interest due to their extensive interaction with electronic devices, a trend that is consistently increasing, demonstrated by the correlation between increased screen time exposure and positive ASRS scores and anxiety symptoms.

The current investigation is not free of limitations. The findings of this research were derived from observational, cross-sectional data, which limits the capacity to establish causal relationships. It is important to note that the design of our study includes online recruitment, and we are unable to eliminate the possibility that students who identified as having ADHD symptoms had better adherence in our research. In other words, the high prevalence reported could be influenced by recruitment bias, and, to avoid this bias, future research should consider recruiting in classroom settings. While we removed students previously diagnosed with ADHD, we did not control for potential cases of ADHD within the family. The heritability of ADHD accounts for up to 70.0% of the observed cases, necessitating rigorous control of this factor in future research initiatives. Finally, our study used self-reported questionnaires and exclusively involved medical students, which precludes extrapolation to other university students, and was conducted on digital platforms, potentially including students who are more engaged with digital media and who, consequently, may have been overrepresented in the sample. However, in the current study, a connection was observed between symptoms associated with ADHD, screen time, and symptoms of anxiety in the college student population. This underscores the need for comprehensive strategies, considering several aspects of students’ daily schedules.

## Conclusion

Medical students show a high prevalence of positive ASRS scores and higher screen time, as well as increased symptoms of anxiety. In addition, we found that screen time was a significant and independent predictor of ASRS scores. These findings highlight the relevance of developing focused interventions designed to regulate screen usage and cultivate healthy technological habits among medical students.

## Availability of Data and Materials

The raw data supporting the conclusions of this article are available from the corresponding author on reasonable request.

## Supplementary Material

Supplementary material associated with this article can be found, in the online version, at https://doi.org/10.62641/aep.v53i3.1892.
