# Supplementary material for: Positive ADHD Scores are Associated With Higher Screen Time and Anxiety Symptoms in Medical Students: Cross-sectional Study
Source: Actas Esp Psiquiatr. 2025 May 5;53(3):494–503. doi: 10.62641/aep.v53i3.1892 (PMC12069922; doi:10.62641/aep.v53i3.1892)
Supplement: Supplementary file 1 [file ActEsp-53-3-494-503-s1.docx]

Supplementary File of: Positive ADHD scores are associated with more screen time and anxiety symptoms in medical students: cross-sectional study - Julia Sader Neves Ferreira, Roberta Molaz da Silva, Carolina Fauzi Hamuche, Rafael Bonfim do Nascimento, Ana Paula Ribeiro, Saulo Gil, Lucas Melo Neves

**Consent form**

You are being invited as a volunteer to participate in the research “THE INFLUENCE OF SCREEN TIME ON SELF-PERCEIVED ATTENTION OF MEDICAL STUDENTS: A CROSS-CUTTING STUDY”. We ask for your authorization to collect and use your responses to the questionnaire below. The use of your responses is linked only to this research project or if you agree to other future ones. In this research we intend to investigate whether high levels of exposure to time of screen influence self-perception of attention problems of medical students. For this research we will collect the responses to the questionnaire below on the Google Forms platform and then compare the results using Microsoft Excel spreadsheets and data analysis programs (a specific program, used only by the researchers involved - SPSS). The risk involved in the research is practically zero because it is an online questionnaire. However, because it involves personal questions, it can cause certain discomfort to those involved. search will contribute to: to analyze if there is one relationship enter at the longest time of screen and the prejudice in the attention of the students. AND like this, to understand better the aspects that directly impact their lives and generate solutions so that the daily lives of these individuals become more enjoyable. To participate in this study the Mr. (the) no will have none cost, nor will you receive any financial benefit. Despite this, if damages resulting from this research are identified and proven, you he has assured the right of to search for the researcher in charge who will assist you and clarify your rights and the possibility of compensation, when applicable. You will receive information about the study in any aspect you wish and will be free to participate or refuse to participate. and the any time and without any prejudice, you can withdraw your consent to the storage and use of your responses, with withdrawal valid from the date of formalization. Your participation is voluntary, and the refusal in to participate no will entail any penalty or modification in the manner in which Mr.

(a) is assisted by the researcher, who will treat your identity with professional standards of confidentiality. The results obtained from the research, based on your responses, will be made available to you when it is completed. Your name or material indicating your participation will not be it will be released without your permission. You will not be identified. in none publication what may result of this research. This term of consent it will be signed electronically. You data, materials and instruments used in the research will be archived with the researcher responsible for a period of 5 (five) years and after that time they will be destroyed. The researchers will treat the your identity with professional standards of confidentiality, complying with Brazilian legislation (Resolutions No. ws /12; 441/11 and Ordinance 2,201 of the National Health Council and its complementary ones), using the information only for academic purposes and scientific. One via of your The responses and this consent form will be sent to your email address at the end of the questionnaire. It is recommended that you save the email containing this copy or print it. Name of the responsible researcher: Lucas Melo Alves. Address: Universidade Santo Amaro – UNISA, Rua Prof. Enéas de Siqueira Neto, 340 - Jardim da Imbuias, São Paulo, SP. Contact of the researcher: email: [lucasmeloneves@uol.com.br,](mailto:lucasmeloneves@uol.com.br) cell phone: (11) 94573-3515, São Paulo, 05/24/2022. Name of participant: _________________________________________ Doc.Identification:________________ Signed: _________________________ I declare what I have appropriately and voluntarily obtained the Free and Informed Consent of this participant to participate in this study, as recommended by Resolution CNS 466, of 12 of December 2012, IV.3 to 6. Prof. Dr. Lucas Melo Neves and Julia Sader Neves Ferreira (student) Date 05/24/2022. By clicking on the button: "I agree in to participate from the search" the Madam) agree to participate in the research under the terms of this TCLE. If you do not agree to participate, simply close this page in your browser.

**Questionnaire demographic and academic characteristics**

What semester of the Medicine course are you in?

1. 2. 3. 4. 5. 6. 7. 8. 9. 10. 11. 12

Date of birth (e.g. 01/01/1990)________________

Sex

1. Female

2. Male

How do you consider yourself:

1. White

2. Yellow

3. Brown

4. Black

5. Indigenous

Height ____________

Weight____________

Do you have a previous diagnosis of ADHD (Attention Deficit Hyperactivity Disorder)?

1. Yes
2. No

**Self-reported questionnaires about ADHD symptoms (ASRS 1.1)**

Select the alternative that best describes how you have felt and behaved in the last 6 months.

1- How often do you have trouble finishing the last details of a task after you have already done the most complicated parts?

Never Rarely Sometimes Frequently Very often

2- How often do you have trouble keeping things in order when you have to perform a task that requires organization?

Never Rarely Sometimes Frequently Very often

3- How often do you have trouble remembering appointments or obligations?

Never Rarely Sometimes Frequently Very often

4- When you have to perform a task that requires a lot of concentration, how often do you avoid or delay starting it?

Never Rarely Sometimes Frequently Very often

5- How often do you fidget in your chair or bounce your hands or feet when you have to sit for a long period of time?

Never Rarely Sometimes Frequently Very often

6- How often do you feel overly active and compelled to do things, as if you were driven by a motor?

Never Rarely Sometimes Frequently Very often

**Sedentary behavior and physical activity time (International Physical Activity Questionnaire - IPAQ)**

We are interested in knowing what types of physical activity people do as part of their daily lives. The questions are related to the amount of time you spend doing physical activity in the PAST week, including today . Please answer each question even if you consider yourself not to be active.

VIGOROUS physical activities are those that require great physical effort and that make you breathe MUCH harder than normal. MODERATE physical activities are those that require some physical effort and that make you breathe A LITTLE harder than normal.

1a - On how many days in the last week did you WALK for at least 10 continuous minutes at home or at work, as a form of transportation to get from one place to another, for leisure, for pleasure or as a form of exercise?

1b On days when you walked for at least 10 continuous minutes, how much time in total did you spend walking each day?

2a. On how many days in the last week did you perform MODERATE activities for at least 10 continuous minutes, such as cycling lightly, swimming, dancing, doing light aerobics, playing recreational volleyball, carrying light weights, doing housework in the house, yard or garden such as sweeping, vacuuming, gardening, or any activity that moderately increased your breathing or heart rate (PLEASE DO NOT INCLUDE WALKING)

2b. On days when you did these moderate activities for at least 10 continuous minutes, how much time in total did you spend doing these activities per day?

3a - On how many days in the last week did you perform VIGOROUS activities for at least 10 continuous minutes, such as running, doing aerobics, playing soccer, cycling fast, playing basketball, doing heavy housework at home, in the yard or digging in the garden, carrying heavy weights or any activity that made your breathing or heart rate increase A LOT.

4a. On average, how many HOURS per day do you spend sitting (considering commuting time, studying at and outside university, leisure time, etc.)?

**Anxiety symptoms (Beck Anxiety Inventory- BAI)**

Below is a list of common anxiety symptoms. Please read each item on the list carefully. Identify how much you have been bothered by each symptom during  the past week , including today , by checking the box next to each symptom.

1. **Numbness or tingling**

0 - Absolutely not; 1 - Slightly - It didn't bother me Much; 2 - Moderately - It was very unpleasant but I could bear it; 3 - Severely - I could hardly bear it;

1. **Feeling hot**

0 - Absolutely not; 1 - Slightly - It didn't bother me Much; 2 - Moderately - It was very unpleasant but I could bear it; 3 - Severely - I could hardly bear it;

1. **Trembling in the legs**

0 - Absolutely not; 1 - Slightly - It didn't bother me Much; 2 - Moderately - It was very unpleasant but I could bear it; 3 - Severely - I could hardly bear it;

1. **Inability to relax**

0 - Absolutely not; 1 - Slightly - It didn't bother me Much; 2 - Moderately - It was very unpleasant but I could bear it; 3 - Severely - I could hardly bear it;

1. **Fear that the worst will happen**

0 - Absolutely not; 1 - Slightly - It didn't bother me Much; 2 - Moderately - It was very unpleasant but I could bear it; 3 - Severely - I could hardly bear it;

1. **Dizziness or lightheadedness**

0 - Absolutely not; 1 - Slightly - It didn't bother me Much; 2 - Moderately - It was very unpleasant but I could bear it; 3 - Severely - I could hardly bear it;

1. **Heart palpitations or racing**

0 - Absolutely not; 1 - Slightly - It didn't bother me Much; 2 - Moderately - It was very unpleasant but I could bear it; 3 - Severely - I could hardly bear it;

1. **Off balance**

0 - Absolutely not; 1 - Slightly - It didn't bother me Much; 2 - Moderately - It was very unpleasant but I could bear it; 3 - Severely - I could hardly bear it;

1. **Terrified**

0 - Absolutely not; 1 - Slightly - It didn't bother me Much; 2 - Moderately - It was very unpleasant but I could bear it; 3 - Severely - I could hardly bear it;

1. **Nervous**

0 - Absolutely not; 1 - Slightly - It didn't bother me Much; 2 - Moderately - It was very unpleasant but I could bear it; 3 - Severely - I could hardly bear it;

1. **Feeling like you're choking**

0 - Absolutely not; 1 - Slightly - It didn't bother me Much; 2 - Moderately - It was very unpleasant but I could bear it; 3 - Severely - I could hardly bear it;

1. **Hands shaking**

0 - Absolutely not; 1 - Slightly - It didn't bother me Much; 2 - Moderately - It was very unpleasant but I could bear it; 3 - Severely - I could hardly bear it;

1. **Trembling**

0 - Absolutely not; 1 - Slightly - It didn't bother me Much; 2 - Moderately - It was very unpleasant but I could bear it; 3 - Severely - I could hardly bear it;

1. **Fear of losing control**

0 - Absolutely not; 1 - Slightly - It didn't bother me Much; 2 - Moderately - It was very unpleasant but I could bear it; 3 - Severely - I could hardly bear it;

1. **Difficulty breathing**

0 - Absolutely not; 1 - Slightly - It didn't bother me Much; 2 - Moderately - It was very unpleasant but I could bear it; 3 - Severely - I could hardly bear it;

1. **Fear of dying**

0 - Absolutely not; 1 - Slightly - It didn't bother me Much; 2 - Moderately - It was very unpleasant but I could bear it; 3 - Severely - I could hardly bear it;

1. **Frightened**

0 - Absolutely not; 1 - Slightly - It didn't bother me Much; 2 - Moderately - It was very unpleasant but I could bear it; 3 - Severely - I could hardly bear it;

1. **Indigestion or abdominal discomfort**

0 - Absolutely not; 1 - Slightly - It didn't bother me Much; 2 - Moderately - It was very unpleasant but I could bear it; 3 - Severely - I could hardly bear it;

1. **Feeling like you might faint**

0 - Absolutely not; 1 - Slightly - It didn't bother me Much; 2 - Moderately - It was very unpleasant but I could bear it; 3 - Severely - I could hardly bear it;

1. **Flushing face**

0 - Absolutely not; 1 - Slightly - It didn't bother me Much; 2 - Moderately - It was very unpleasant but I could bear it; 3 - Severely - I could hardly bear it;

1. **Sweating (not due to heat)**

0 - Absolutely not; 1 - Slightly - It didn't bother me Much; 2 - Moderately - It was very unpleasant but I could bear it; 3 - Severely - I could hardly bear it;
